# Primary aldosteronism and lower-extremity arterial disease: a two-sample Mendelian randomization study

**DOI:** 10.1186/s12933-023-02086-x

**Published:** 2023-12-20

**Authors:** Jinbo Hu, Qinglian Zeng, Xiangjun Chen, Wenjin Luo, Ziwei Tang, Mei Mei, Wenrui Zhao, Zhipeng Du, Zhiping Liu, Qifu Li, Qingfeng Cheng, Shumin Yang

**Affiliations:** Department of Endocrinology, the First Affiliated Hospital of Chongqing Medical University, No. 1 Youyi St, Chongqing, 400016 China

**Keywords:** Primary aldosteronism, Lower-extremity arterial disease, Diabetes, Mendelian randomization, Causal relationship

## Abstract

**Background and Aims:**

Primary aldosteronism (PA) is an adrenal disorder of autonomous aldosterone secretion which promotes arterial injury. We aimed to explore whether PA is causally associated with lower-extremity arterial disease (LEAD).

**Methods:**

We included 39,713 patients with diabetes and 419,312 participants without diabetes from UK Biobank. We derived a polygenic risk score (PRS) for PA based on previous genome-wide association studies (GWAS). Outcomes included LEAD and LEAD related gangrene or amputation. We conducted a two-sample Mendelian randomization analysis for PA and outcomes to explore their potential causal relationship.

**Results:**

In whole population, individuals with a higher PA PRS had an increased risk of LEAD. Among patients with diabetes, compared to the subjects in the first tertile of PA PRS, subjects in the third tertile showed a 1.24-fold higher risk of LEAD (OR 1.24, 95% CI 1.03–1.49) and a 2.09-fold higher risk of gangrene (OR 2.09, 95% CI 1.27–3.44), and 1.72-fold higher risk of amputation (OR 1.72, 95% CI 1.10–2.67). Among subjects without diabetes, there was no significant association between PA PRS and LEAD, gangrene or amputation. Two-sample Mendelian randomization analysis indicated that genetically predictors of PA was significantly associated with higher risks of LEAD and gangrene (inverse variance weighted OR 1.20 [95% CI 1.08–1.34]) for LEAD, 1.48 [95% CI 1.28–1.70] for gangrene), with no evidence of significant heterogeneity or directional pleiotropy.

**Conclusions:**

Primary aldosteronism is genetically and causally associated with higher risks of LEAD and gangrene, especially among patients with diabetes. Targeting on the autonomous aldosterone secretion may prevent LEAD progression.

**Supplementary Information:**

The online version contains supplementary material available at 10.1186/s12933-023-02086-x.

## Introduction

Lower-extremity arterial disease (LEAD), also named as lower extremity peripheral artery disease, is an atherosclerotic disease of the arteries supplying the legs [1] LEAD affects approximately 230 million adults worldwide, and is associated with high risks of cardiovascular events, gangrene and amputation [1–3], especially for patients with diabetes [4]. Despite the high prevalence and detrimental impact on human health, LEAD has been understudied when compared with other atherosclerotic cardiovascular diseases such as stroke and coronary heart disease [1, 3], and treatment options for LEAD are still substantial unmet needs [5].

Primary aldosteronism, a high prevalence but largely unrecognized disease [6], is an autonomous hypersecretion of aldosterone from the adrenal cortex, leading to an increase in plasma aldosterone concentration and suppression of renin [7–9]. Compared to individuals with essential hypertension or normotensive, patients with primary aldosteronism had increased levels of artery intima-media thickness and pulse-wave velocity [10], which were shown to be LEAD related parameters [11–13]. A decreased brachial artery flow-mediated dilation (another LEAD related parameter [14, 15]) was also observed among participants with high aldosterone-to-renin ratio (ARR, an indicator of subtle primary aldosteronism) when compared to individuals who had normal ARR [16]. These observational data implied that primary aldosteronism may be a crucial risk factor for LEAD, although there is no study exploring the causal relationship between primary aldosteronism and the LEAD progression.

The Mendelian randomization is an useful method to assess the potential causal relationship by excluding observational confounders for both exposures and outcomes [17–19]. In the current study, we systematically reviewed the published data on the genome-wide association studies (GWAS) of primary aldosteronism and derived a polygenic risk score (PRS) for the primary aldosteronism. Then we conducted a two-sample mendelian randomization study to explore whether primary aldosteronism is genetically and causally associated with LEAD progression.

## Methods

### Study population

The UK Biobank is a prospective cohort study that recruited 502,664 participants aged 40–69 years, from England, Scotland, and Wales between 2006 and 2010. The current analysis was approved by UK Biobank (application identification: 66536). This analysis received approval from the National Information Governance Board for Health and Social Care and the National Health Service North West Multicenter Research Ethics Committee (reference 13/NW/0382). All participants provided informed consent through electronic signature at the first assessment.

In this analysis, we included Caucasian participants who were unrelated and whose information of genotype was available. The central quality control and genotype imputation for genotype data have been performed by UK Biobank. Database for the current analysis contains genome-wide genotyping of baseline samples from all participants, clinical examinations, assays of biological samples, and is supplemented by linkage with electronic records of diseases.

### GWAS data for primary aldosteronism

We systematically searched GWAS data regarding primary aldosteronism via PubMed/MEDLINE and EMBASE databases through November 13th, 2022. We used a combination of free text and subheadings from MeSH and EMTREE terms, and only one study was available with GWAS data [20]. In the included study, primary aldosteronism was defined according to the Endocrine Society guidelines [20, 21]. We selected genetic loci if they were significantly associated with primary aldosteronism in GWAS (*P* ≤ 10 × 10^–6^), with minor allele frequencies > 0.10 in European population, and part of these were demonstrated to be functional on autonomous aldosterone secretion in vitro and/or in vivo. In order to confirm the candidate gene loci are functional for primary aldosteronism, we only included genes which directly regulates the production of adrenal aldosterone and is not mediated by renin or angiotensin.

Finally, we included a total of 22 phenotype-associated single Nucleotide Polymorphisms (SNPs) regulating autonomous aldosterone secretion. We extracted genotypes for the phenotype-associated SNP from UK biobank.

### Assessment of lower extremity arterial disease

The lower extremity arterial disease (LEAD) was diagnosed if individual had femoral artery surgery or severe intermittent claudication. We defined femoral artery surgery as bypass, endarterectomy and angioplasty of femoral artery (Office of Population Censuses and Surveys Classification of Interventions and Procedures, version 4 [OPCS4]: L591, L592, L593, L594, L595, L596, L597, L598, L599, L601, L602, L603, L604, L631). We defined intermittent claudication based on the self-reported intermittent claudication (Data-Field 20002, code 1087) at baseline, or online questionnaire derived from the WHO/Rose Questionnaire on intermittent claudication [22] which included all the following seven criteria: presence of leg pain on walking (field ID 4728), leg pain in calf/calves (field ID 5463), leg pain when walking uphill or hurrying (field ID 5474), pain usually disappears in less than 10 min (field ID 5518), stop or slow down when get pain in walking (field ID 5507), absence of leg pain when standing still or sitting (field ID 5452), leg pain when walking ever disappears while walking (field ID 5496). We further defined the severe intermittent claudication as a leg pain when walking normally (field ID 5485).

We defined LEAD progression as LEAD related gangrene or amputation. The gangrene was defined as the death of tissue due to the lack of blood flow (ICD10: R02, I7021). We defined amputation as the loss or removal of a toe, foot, or leg (OPCS4: X091, X092, X093, X094, X095, X098, X099, X101, X102, X103, X104, X108, X109, X111, X112, X118, X119, W151, W152, W153, W154, W155, W156, W157, W158, W159; field ID 5540). In the current analysis, we restrained the gangrene or amputation to the lower extremities among patients with LEAD. For patients who experienced multiple events of LEAD, we included them for each outcome analysis.

### Polygenic risk score of primary aldosteronism

The PA PRS were calculated as the sum of 22 weight scores obtained by risk alleles multiplied by the β corresponding to 22 significantly associated SNP. We listed a total of 22 PA related SNPs in Additional file 1: Table S1.

### Statistical analysis

We used descriptive variables to summarize individual characteristics by the tertiles of PA PRS. A continuous variable with a normal distribution was presented as a mean ± standard deviation, and a categorical variable was presented as percent. The missing rate of covariates was less than 5.0%.

We developed multivariable adjusted models based on restricted cubic spline regression to evaluate linear or nonlinear associations between PA PRS and outcomes (including LEAD and LEAD related gangrene or amputation). We calculated the odds ratios (ORs) and 95% confidence intervals (CIs) based on a logistic regression model to analyze the relationship between PA PRS and above outcomes. In the multivariable models, we adjusted for age, sex, PCA10, genotyping batch and UK Biobank assessment centre. We used R version 4.0.3 for data analyses on the supercomputer platform (inspur M5), and a two-tailed P value < 0.05 was considered statistically significant.

### Two-sample Mendelian randomization

In order to investigate the potential causal relationship between primary aldosteronism and LEAD, we performed a two-sample Mendelian randomization based on the summary-level data from public genome-wide association studies [23, 24]. We included a reported GWAS for primary aldosteronism which was performed among participants of European ancestry [20]. We also included GWAS data for LEAD and gangrene which was also conducted among individuals of European ancestry [24]. We selected loci with *P* < 10 × 10^–6^ and minor allele frequencies > 0.30 in European population in the reported GWAS. We used Mendelian randomization pleiotropy residual sum and outlier (MR-PRESSO) to identify the horizontal pleiotropic outlier. All SNPs were available in the outcome GWAS summary statistics. To ensure that the significant SNPs were unique to primary aldosteronism, the genetic instrument was checked for overlap with body mass index, glycated hemoglobin, low density lipoprotein cholesterol and smoking status.

We conducted the two-sample Mendelian randomization analysis by using “TwoSampleMR” with R package version 0.5.5. Four complementary methods, namely inverse variance weighted, mendelian randomisation-Egger (MR-Egger), weighted median, and weighted mode based estimation, were used to make different assumptions about horizontal pleiotropy. A consistent effect across four methods is less likely to be a false positive [25]. We used the MR-Egger intercept and MR-PRESSO (Mendelian randomization pleiotropy residual sum and outlier) to test the presence of directional pleiotropy. We assessed the presence of heterogeneity between individual SNP effect estimates by using Cochrane’s Q test for inverse variance weighted analyses and Rücker’s Q test for MR-Egger analyses. In addition, we performed a leave-one-out analysis to evaluate the influence of outlying or pleiotropic SNPs [26].

## Results

### Demographic and biochemical characteristics

A total of 459025 UK Biobank participants with complete data met the inclusion criteria were included in this study. We summarized participants’ characteristics according to the PA PRS tertiles (Table 1). Among 39,713 patients who were diagnosed as diabetes, 684 patients had LEAD, 115 patients had lower limb gangrene due to LEAD, and 120 patients had lower limb amputation due to LEAD. Diabetic patients with amputation and gangrene had a significantly higher PA PRS compared to those without (Additional file 1: Figure S1). The PA PRS is 11.90 ± 4.22 for patients with diabetes, and 11.83 ± 4.22 for patients without diabetes.

**Table 1:** Baseline characteristic of participants according to the tertiles of PA PRS

	Individuals with diabetes			Individuals without diabetes		
	0.80–10.10	10.10–13.88	13.88–23.13	0–10.03	10.03–13.82	13.82–23.14
Number	13105	13503	13105	138373	142566	138,373
Age (years)	59.77 (7.09)	59.51 (7.14)	59.70 (7.11)	56.50 (8.04)	56.48 (8.06)	56.49 (8.05)
Female, number (%)	7783 (59.4)	8085 (59.9)	7883 (60.2)	61337 (44.3)	63420 (44.5)	61,339 (44.3)
Smoking, number (%)	1615 (12.3)	1774 (13.1)	1694 (12.9)	14130 (10.2)	14541 (10.2)	14,121 (10.2)
Body Mass Index (kg/m^2^)	31.44 (5.73)	31.45 (5.79)	31.42 (5.77)	27.00 (4.46)	27.00 (4.48)	27.04 (4.50)
Total Cholesterol (mmol/L)	4.98 (1.25)	4.99 (1.25)	4.97 (1.24)	5.79 (1.11)	5.78 (1.11)	5.78 (1.11)
Triglycerides (mmol/L)	2.27 (1.31)	2.26 (1.28)	2.25 (1.26)	1.71 (0.99)	1.70 (0.98)	1.70 (0.98)
HDL-C (mmol/L)	1.22 (0.33)	1.22 (0.33)	1.23 (0.33)	1.48 (0.38)	1.47 (0.38)	1.47 (0.38)
LDL-C (mmol/L)	3.06 (0.94)	3.07 (0.93)	3.06 (0.93)	3.62 (0.85)	3.61 (0.85)	3.61 (0.85)
HbAlc (%)	6.52 (1.20)	6.54 (1.23)	6.52 (1.20)	5.34 (0.35)	5.34 (0.35)	5.34 (0.37)
SBP (mmHg)	144.53 (18.98)	144.58 (19.35)	145.02 (19.13)	139.21 (19.50)	139.43 (19.67)	139.68 (19.76)
DBP (mmHg)	82.75 (10.82)	83.10 (10.86)	82.95 (10.80)	82.00 (10.66)	82.11 (10.63)	82.18 (10.68)
LEAD, number (%)	201 (1.5)	235 (1.7)	248 (1.9)	336 (0.2)	403 (0.3)	348 (0.3)
Gangrene, number (%)	23 (0.2)	44 (0.3)	48 (0.4)	20 (0.01)	14 (0.01)	15 (0.01)
Amputation, number (%)	31 (0.2)	36 (0.3)	53 (0.4)	38 (0.02)	26 (0.02)	25 (0.02)

*Tertile* tertiles of PA PRS, *PA* primary aldosteronism, *PRS* polygenic risk scores, *HDL-C* high density lipoprotein cholesterol, *LDL-C* low density lipoprotein cholesterol, *HbAlc* glycated haemoglobin, *SBP* Systolic Blood Pressure, *DBP* Diastolic Blood Pressure, *LEAD* Lower Extremity Arterial Disease

### Association between PA PRS and outcomes

**Fig. 1:**
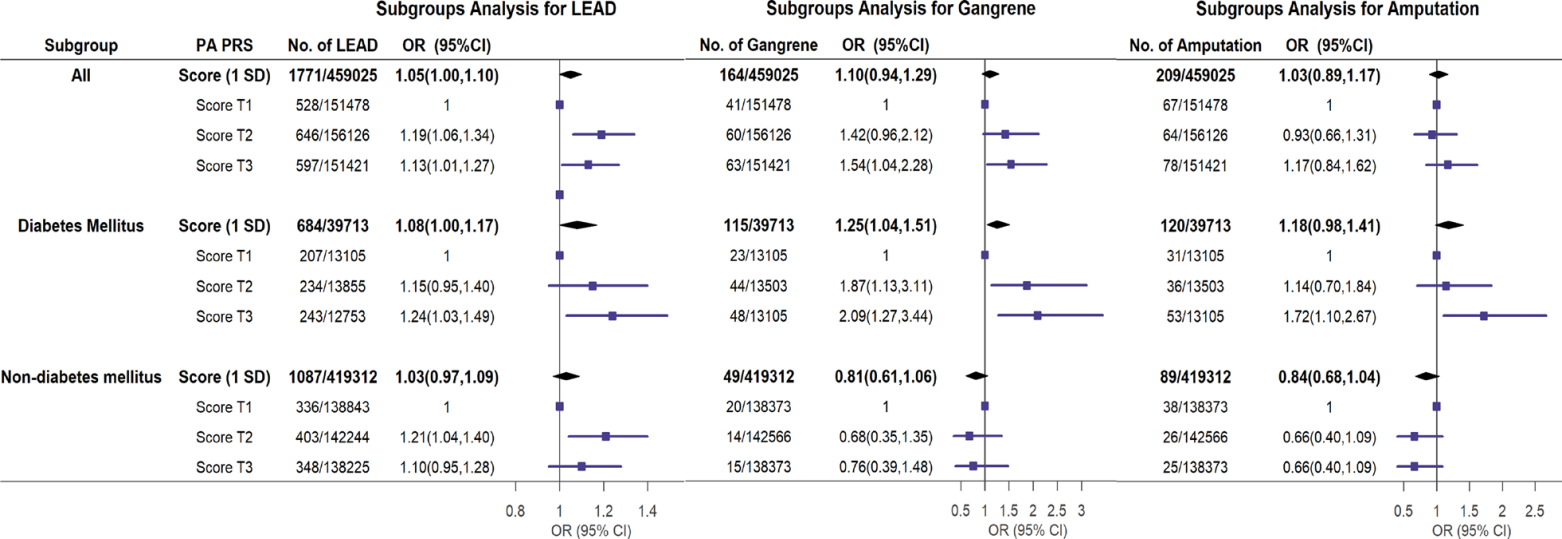
Forest plots of the multivariate regression analysis for PA PRS on lower extremity arterial disease (LEAD), gangrene, amputation. The subgroup analyses were conducted in diabetes and non-diabetes. Models were adjusted for age, sex, 10 genotype principal components (PCs), genotyping batch and assessment centre. *PA* primary aldosteronis, *PRS* polygenic risk score

In the whole population, when compared to those in the first tertile of PA PRS, subjects in the third tertile showed a 1.13-fold higher risk of LEAD (OR 1.13, 95% CI 1.01–1.27) and a 1.54-fold higher risk of gangrene (OR 1.54, 95%CI 1.04–2.28), while the risk of amputation was not changed. Among patients with diabetes, one-SD increment of PA PRS resulted in a 1.08-fold higher risk of LEAD (OR 1.08, 95% CI 1.00–1.17), a 1.25-fold higher risk of gangrene (OR 1.25, 95% CI 1.04–1.51), while the risk of amputation was not changed. When compared to the subjects in the first tertile of PA PRS, subjects in the third tertile showed a 1.24-fold higher risk of LEAD (OR 1.24, 95% CI 1.03–1.49) and a 2.09-fold higher risk of gangrene (OR 2.09, 95% CI 1.27–3.44), and 1.72-fold higher risk of amputation (OR 1.72, 95% CI 1.10–2.67) (Fig. 1). However, among subjects without diabetes, the association between PA PRS and LEAD, gangrene or amputation was not significant (Fig. 1). Further results for the association between individual SNPs and LEAD, gangrene or amputation were summarized in Additional file 1: Table S2.

**Fig. 2:**
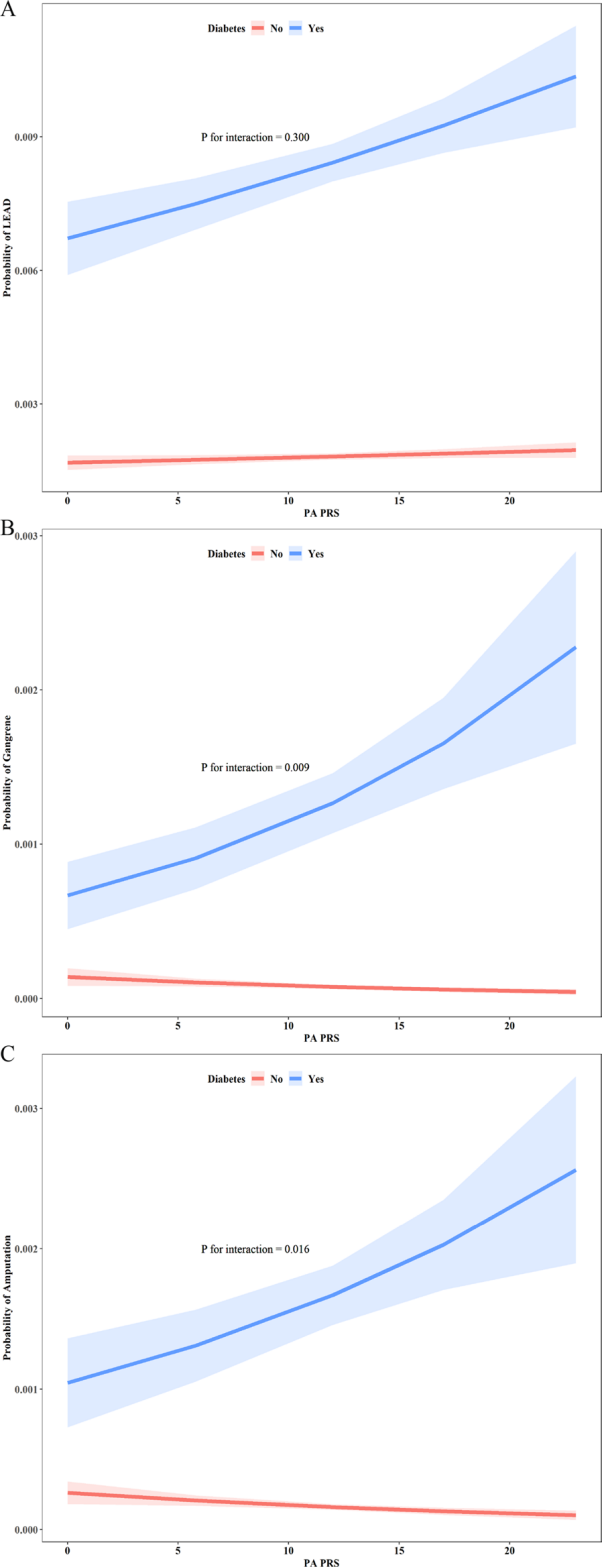
Association between PA PRS and proportions of lower extremity arterial disease (LEAD) (Panel **A**), gangrene (Panel **B**) and amputation (Panel **C**), stratified by diabetes. *PA* primary aldosteronism, *PRS* polygenic risk score

The association between PA PRS and risk of LEAD, gangrene and amputation were further analyzed by stratification of diabetes in whole patients (Fig. 2). Compared to patients without diabetes, risk of LEAD, gangrene and amputation increased more obviously with the increase of PA PRS in patients with diabetes. And there were interaction effect between PA PRS and diabetes for gangrene and amputation.

### Two-sample Mendelian randomized analysis

**Fig. 3:**
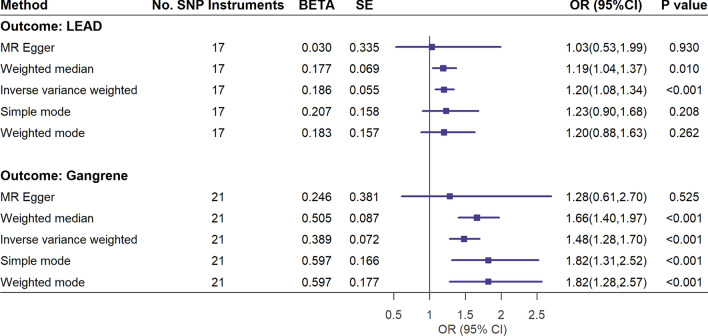
Forest plots of the Two-sample Mendelian randomization analysis for primary aldosteronism on lower extremity arterial disease and gangrene. Two-sample Mendelian randomization for LEAD remove rs2137320, rs4980379, rs569016, rs587961, rs661348; two-sample Mendelian randomization for gangrene remove rs6679531

Most of the loci included as the instrument variables were not associated with common risk factors for LEAD and gangrene, including body mass index, glycated hemoglobin, smoking and low-density lipoprotein cholesterol (Additional file 1: Table S3). Two-Sample Mendelian randomized analysis suggested a causal relationship between primary aldosteronism and LEAD (inverse variance weighted OR 1.20 [95% CI 1.08–1.34], weighted median OR 1.19 [95% CI 1.04–1.37]). There was a causal relationship between primary aldosteronism and gangrene (inverse variance weighted OR 1.48 [95% CI 1.28–1.70], weighted median OR 1.66 [95% CI 1.40–1.97], simple mode OR 1.82 [95% CI 1.31–2.52], weighted mode OR 1.82 [95% CI 1.28–2.57])** (**Fig. 3). Additional file 1: Table S4 summarized GWAS consortiums used for individual phenotype of the included SNPs. Additional file 1: Table S5 showed the Two-Sample Mendelian randomization results for associations between individual SNPs and LEAD and gangrene. There were no evidence of pleiotropy and heterogeneity for the association between renin-independent aldosteronism and LEAD and gangrene (Additional file 1: Table S6). Further leave-one-out sensitivity analysis for Mendelian randomization also showed robust causal relationship between renin-independent aldosteronism and LEAD (Additional file 1: Figure S2) or gangrene. (Additional file 1: Figure S3).

## Discussion

Our study firstly demonstrated that a higher PA PRS was significantly associated with increased risks of LEAD, gangrene and amputation, especially for patients with diabetes and poorly controlled glycemia. Importantly, the two-sample Mendelian randomization analysis indicated that primary aldosteronism was causally associated with LEAD and gangrene. These data indicated that targeting on the autonomous aldosterone secretion may be a treatment option for LEAD, especially for diabetic patients.

Several studies explored the relationship between primary aldosteronism and LEAD related parameters, including a higher pulse-wave velocity, an increased peripheral arterial wall inflammation, and a decreased flow-mediated dilation. A systematic review included 12 studies and pooled 445 cases of primary aldosteronism and 472 controls of essential hypertension or normotensive subjects. Compared to subjects with controls, patients with primary aldosteronism showed a higher common carotid artery intima-media thickness and a higher pulse-wave velocity [10]. Another case–control study recruited 15 cases of primary aldosteronism and 15 age-, sex- and blood pressure-matched controls of essential hypertension, and these who had primary aldosteronism showed a significantly increased peripheral arterial wall inflammation [27]. Although these data implied that primary aldosteronism significantly correlated with multiple markers of LEAD, evidence demonstrating the direct association between primary aldosteronism and LEAD related outcome is still absent, and whether the relationship is causal or mediated by other unrecognized confounders remains unclear.

The application of PRS to population study was helpful for clarifying the genetic relationship between exposure and outcome, and the efficacy of PRS was shown to be comparable to monogenic mutations and established clinical risk factors [28–30]. After an integrated analysis of previous GWAS data and experimental data [20, 31, 32], we included 22 loci to calculate the PA PRS. Logistic regression analysis indicated that a higher PA PRS was associated with higher risks of LEAD, gangrene and amputation, especially for patients with diabetes or poorly controlled glycemia. One explanation for the necessity of a concurrent diabetic state is the synergistic amplification effect of hyperglycemia and excessive aldosterone on the vascular damage. High glucose stimulates mineralocorticoid receptor transcriptional activity and leads to the stabilization of mineralocorticoid receptor [33], which enhance the sensitivity to aldosterone-induced vascular injury [34, 35]. Another explanation may be embedded in the interactions among obesity, insulin resistance and vascular mineralocorticoid receptor [36]. As an important feature of type 2 diabetes, obesity has been shown to significantly induce cardiovascular injury which depend on the activation of endothelial mineralocorticoid receptor [37]. Furthermore, multifactorial etiologies in patients with diabetes, such as propensity for wound formation, prolonged wound healing and disorganized immune system [38], are inclined to exert synergies between primary aldosteronism and diabetes to promote the progression of LEAD, particularly for gangrene and amputation.

The Two-sample Mendelian randomization is an useful method to assess the potential causal relationship by excluding observational confounders for both exposures and outcomes [17–19]. Our current two-sample Mendelian randomization analysis showed that using different methods of Mendelian randomization such as inverse variance weighted and weighted median, genetically predictors of PA was consistently associated with higher risk of LEAD and gangrene, and there was no significant heterogeneity or substantial directional pleiotropy. These data suggested that primary aldosteronism may be causally associated with LEAD related outcome.

Treatment options for LEAD are unmet needs, and the potential causal relationship between primary aldosteronism and LEAD might lighten a new clue for preventing LEAD progression. For patients with primary aldosteronism, either adrenalectomy or mineralocorticoid receptor antagonist was shown to significantly reduce the brachial-ankle pulse wave velocity [39, 40]. Spironolactone and eplerenone, classical mineralocorticoid receptor antagonists, were shown to significantly reduce the pulse wave velocity among patients with hypertension [41, 42].

The main strengths of the current study included the large sample size and a two-sample Mendelian randomization analysis in UK Biobank. There are several limitations of the current study. Firstly, we did not measure aldosterone or renin in UK Biobank, and screening or confirmatory tests for primary aldosteronism was hardly conducted. Secondly, the number of gangrene or amputation due to LEAD was relatively limited. Thirdly, the cohort of UK Biobank mainly consisted of White, which should be validated in more cohorts of different populations.

In conclusion, primary aldosteronism is genetically and causally associated with a higher risk of LEAD, especially for patients with diabetes. Our analyses support that the mineralocorticoid receptor antagonists might prevent LEAD progression.

### Supplementary Information

**Additional file 1: Table S1.** SNP instruments related with primary aldosteronism. **Table S2.** Relationship between each SNP genotype of primary aldosteronism and LEAD events. **Table S3.** The association of PA PRS and 22 SNPs included as the instrument variables in two-sample Mendelian randomization analysis with the common risk factors related to lower extremity arterial disease, gangrene, amputation. **Table S4.** Description of GWAS consortiums used for each phenotype. **Table S5.** Effect of each SNP on LEAD events. **Table S6.** Tests of pleiotropy and heterogeneity for Mendelian randomization between primary aldosteronism and LEAD events. **Figure S1.** The PA PRS between diabetes patients with gangrene or amputation and patients without gangrene or amputation. PA: primary aldosteronism. PRS: polygenic risk score. **Figure S2.** Leave-one-out sensitivity analysis for mendelian randomization between primary aldosteronism and lower extremity arterial disease. **Figure S3.** Leave-one-out sensitivity analysis for mendelian randomization between primary aldosteronism and gangrene. **Figure S4.** The regression analysis was conducted to estimate the correlation between PA PRS and LEAD, and LEAD-related outcomes after excluding 7 specific SNPs proven insignificant for LEAD or LEAD-related outcomes.

## Data Availability

The individual participant data collected for the current study can not be shared without UK Biobank’s explicit written approval.
