# Supplementary material for: Primary aldosteronism and lower-extremity arterial disease: a two-sample Mendelian randomization study
Source: Cardiovasc Diabetol. 2023 Dec 20;22:352. doi: 10.1186/s12933-023-02086-x (PMC10734059; doi:10.1186/s12933-023-02086-x)
Supplement: Supplementary file 1 — Additional file 1: Table S1. SNP instruments related with primary aldosteronism. Table S2. Relationship between each SNP genotype of primary aldosteronism and LEAD events. Table S3. The association of PA PRS and 22 SNPs included as the instrument variables in two-sample Mendelian randomization analysis with the common risk factors related to lower extremity arterial disease, gangrene, amputation. Table S4. Description of GWAS consortiums used for each phenotype. Table S5. Effect of each SNP on LEAD events. Table S6. Tests of pleiotropy and heterogeneity for Mendelian randomization between primary aldosteronism and LEAD events. Figure S1. The PA PRS between diabetes patients with gangrene or amputation and patients without gangrene or amputation. PA: primary aldosteronism. PRS: polygenic risk score. Figure S2. Leave-one-out sensitivity analysis for mendelian randomization between primary aldosteronism and lower extremity arterial disease. Figure S3. Leave-one-out sensitivity analysis for mendelian randomization between primary aldosteronism and gangrene. Figure S4. The regression analysis was conducted to estimate the correlation between PA PRS and LEAD, and LEAD-related outcomes after excluding 7 specific SNPs proven insignificant for LEAD or LEAD-related outcomes. [file 12933_2023_2086_MOESM1_ESM.docx]

**Additional Table 1. SNP instruments related with primary aldosteronism**

| SNP | REF/ALT | AAF | BETA | SE | P value |
| --- | --- | --- | --- | --- | --- |
| rs284277 | C/A | 0.38 | 0.482 | 0.08 | 3.22E-09 |
| rs880315 | C/T | 0.37 | 0.464 | 0.08 | 1.03E-08 |
| rs6679531 | C/T | 0.36 | 0.761 | 0.14 | 5.25E-08 |
| rs12080303 | G/A | 0.32 | 0.742 | 0.14 | 1.84E-07 |
| rs277129 | C/A | 0.65 | 0.525 | 0.11 | 9.11E-07 |
| rs563854 | T/C | 0.52 | 0.482 | 0.10 | 9.36E-07 |
| rs1869799 | T/C | 0.63 | 0.476 | 0.08 | 6.12E-09 |
| rs1200470 | T/C | 0.52 | 0.482 | 0.10 | 8.43E-07 |
| rs1535532 | T/C | 0.63 | 0.507 | 0.08 | 5.76E-10 |
| rs1902272 | T/C | 0.69 | 0.451 | 0.09 | 1.10E-07 |
| rs277143 | G/A | 0.51 | 0.489 | 0.10 | 4.82E-07 |
| rs1671966 | T/C | 0.6 | 0.604 | 0.10 | 3.95E-09 |
| rs1327981 | G/T | 0.51 | 0.507 | 0.10 | 1.42E-07 |
| rs1327980 | C/T | 0.5 | 0.519 | 0.10 | 8.11E-08 |
| rs9603395 | C/A | 0.67 | 0.571 | 0.11 | 1.51E-07 |
| rs2025908 | A/G | 0.49 | 0.536 | 0.10 | 4.30E-08 |
| rs1571303 | T/C | 0.49 | 0.501 | 0.10 | 2.58E-07 |
| rs569016 | T/C | 0.75 | 0.793 | 0.16 | 7.38E-07 |
| rs587961 | T/C | 0.67 | 0.412 | 0.08 | 8.67E-07 |
| rs2137320 | G/A | 0.42 | 0.412 | 0.08 | 1.92E-07 |
| rs4980379 | C/T | 0.4 | 0.470 | 0.1 | 8.78E-07 |
| rs661348 | T/C | 0.45 | 0.392 | 0.08 | 5.75E-07 |

SNP, single nucleotide polymorphism; ALT, alternative allele; REF, reference allele; AAF, alternative allele frequency; SE, standard error.

**Additional Table 2. Relationship between each SNP genotype of primary aldosteronism and LEAD events**

|  | LEAD/Gangrene/Amputation/Number | |  | LEAD | |  | Gangrene | |  | Amputation | |
| --- | --- | --- | --- | --- | --- | --- | --- | --- | --- | --- | --- |
|  | Wildtype (ref) | Homozygosity |  | OR (95% CI) | *p* value |  | OR (95% CI) | *p* value |  | OR (95% CI) | *p* value |
| rs284277 | 254/42/43/15353 | 70/12/12/4480 |  | 1.01(0.90,1.14) | 0.827 |  | 1.04(0.78,1.37) | 0.804 |  | 1.03(0.78,1.36) | 0.827 |
| rs880315 | 280/46/51/15955 | 64/12/9/3974 |  | 0.97(0.86,1.09) | 0.571 |  | 1.02(0.76,1.36) | 0.893 |  | 0.90(0.67,1.19) | 0.451 |
| rs6679531 | 242/41/50/14952 | 74/14/13/3459 |  | 1.15(1.02,1.30) | 0.022 |  | 1.19(0.89,1.59) | 0.248 |  | 0.98(0.73,1.31) | 0.893 |
| rs12080303 | 263/46/54/16664 | 74/14/14/3066 |  | 1.22(1.08,1.38) | 0.001 |  | 1.19(0.89,1.60) | 0.238 |  | 1.05(0.78,1.40) | 0.754 |
| rs277129 | 74/6/7/4445 | 293/59/56/16626 |  | 1.03(0.92,1.15) | 0.638 |  | 1.49(1.10,2.01) | 0.011 |  | 1.32(0.98,1.76) | 0.065 |
| rs563854 | 127/14/15/7880 | 158/29/31/8480 |  | 1.08(0.96,1.22) | 0.21 |  | 1.36(1.01,1.84) | 0.043 |  | 1.36(1.02,1.81) | 0.037 |
| rs1869799 | 82/8/10/4899 | 270/50/49/15021 |  | 1.05(0.94,1.18) | 0.389 |  | 1.36(1.01,1.83) | 0.046 |  | 1.19(0.90,1.58) | 0.223 |
| rs1200470 | 154/17/18/9342 | 194/36/41/9851 |  | 1.10(0.99,1.23) | 0.085 |  | 1.39(1.06,1.81) | 0.018 |  | 1.45(1.12,1.89) | 0.005 |
| rs1535532 | 91/9/11/5471 | 285/54/52//15767 |  | 1.05(0.94,1.18) | 0.348 |  | 1.35(1.02,1.79) | 0.038 |  | 1.20(0.92,1.58) | 0.178 |
| rs1902272 | 52/4/5/2692 | 321/62/60/18283 |  | 1.07(0.94,1.22) | 0.289 |  | 1.43(1.02,2.00) | 0.039 |  | 1.29(0.93,1.79) | 0.126 |
| rs277143 | 150/17/17/9138 | 189/35/38/9598 |  | 1.10(0.99,1.23) | 0.084 |  | 1.36(1.04,1.78) | 0.025 |  | 1.44(1.10,1.90) | 0.008 |
| rs1671966 | 93/6/8/5859 | 250/43/43/13702 |  | 1.08(0.96,1.21) | 0.208 |  | 1.34(1.01,1.78) | 0.043 |  | 1.29(0.97,1.71) | 0.08 |
| rs1327981 | 154/18/21/9898 | 176/32/35/9245 |  | 1.11(0.99,1.24) | 0.062 |  | 1.34(1.03,1.75) | 0.032 |  | 1.33(1.02,1.73) | 0.033 |
| rs1327980 | 157/18/21/10018 | 177/32/35/9340 |  | 1.10(0.99,1.23) | 0.077 |  | 1.34(1.03,1.75) | 0.031 |  | 1.33(1.03,1.74) | 0.032 |
| rs9603395 | 60/6/9/3827 | 323/57/56/17677 |  | 1.10(0.97,1.24) | 0.122 |  | 1.30(0.96,1.76) | 0.091 |  | 1.17(0.87,1.57) | 0.289 |
| rs2025908 | 136/13/18/9015 | 132/25/30/7171 |  | 1.11(0.98,1.25) | 0.101 |  | 1.48(1.10,2.00) | 0.01 |  | 1.46(1.09,1.96) | 0.011 |
| rs1571303 | 133/13/16/8153 | 93/18/21/4957 |  | 1.06(0.93,1.22) | 0.386 |  | 1.49(1.06,2.10) | 0.023 |  | 1.50(1.06,2.12) | 0.022 |
| rs569016 | 41/7/10/2269 | 357/56/54/21114 |  | 0.97(0.86,1.11) | 0.667 |  | 0.86(0.64,1.16) | 0.318 |  | 0.73(0.55,0.96) | 0.024 |
| rs587961 | 88/19/11//5112 | 264/41/48/16144 |  | 0.96(0.86,1.07) | 0.479 |  | 0.83(0.64,1.08) | 0.17 |  | 1.07(0.82,1.40) | 0.604 |
| rs2137320 | 260/48/49/14656 | 103/17/16/6114 |  | 0.97(0.87,1.09) | 0.64 |  | 0.89(0.68,1.17) | 0.419 |  | 0.88(0.68,1.15) | 0.355 |
| rs4980379 | 271/50/51/15330 | 84/13/11/4906 |  | 0.99(0.88,1.11) | 0.826 |  | 0.89(0.67,1.18) | 0.418 |  | 0.85(0.64,1.12) | 0.246 |
| rs661348 | 194/33/37/11660 | 117/19/20/6340 |  | 1.06(0.94,1.19) | 0.327 |  | 1.04(0.79,1.37) | 0.791 |  | 0.99(0.76,1.30) | 0.966 |

Age, sex, PCA10, UK Biobank assessment centre and genotyping batch were included as covariates in the regression analyses.

LEAD, lower extremity arterial disease.

**Additional Table 3. The association of PA PRS and 22 SNPs included as the instrument variables in two-sample Mendelian randomization analysis with the common risk factors related to lower extremity arterial disease, gangrene, amputation.**

| SNPs | **Body mass index** | |  | **Glycated hemoglobin** | |  | **low density lipoprotein cholesterol** | |  | **Smoking status** | |
| --- | --- | --- | --- | --- | --- | --- | --- | --- | --- | --- | --- |
|  | Beta | p value |  | Beta | p value |  | Beta | p value |  | OR (95% CI) | p value |
| rs284277 | 0.054 | 0.22 |  | 0.002 | 0.857 |  | -0.007 | 0.33 |  | 0.98 (0.94, 1.03) | 0.402 |
| rs880315 | 0.053 | 0.239 |  | 0 | 0.985 |  | -0.009 | 0.214 |  | 0.98 (0.93, 1.03) | 0.397 |
| rs6679531 | -0.068 | 0.152 |  | 0.011 | 0.278 |  | 0.01 | 0.217 |  | 1.00 (0.96, 1.06) | 0.854 |
| rs12080303 | -0.102 | 0.034 |  | 0.01 | 0.327 |  | 0.012 | 0.115 |  | 1.00 (0.95, 1.05) | 0.942 |
| rs277129 | 0.057 | 0.188 |  | 0.008 | 0.409 |  | 0.004 | 0.549 |  | 1.05 (1.00, 1.09) | 0.056 |
| rs563854 | 0.021 | 0.632 |  | 0.004 | 0.645 |  | -0.008 | 0.3 |  | 1.03 (0.98, 1.08) | 0.198 |
| rs1869799 | 0.024 | 0.577 |  | 0.009 | 0.318 |  | -0.007 | 0.308 |  | 1.03 (0.98, 1.07) | 0.264 |
| rs1200470 | 0.015 | 0.718 |  | 0.004 | 0.644 |  | -0.006 | 0.376 |  | 1.02 (0.98, 1.06) | 0.41 |
| rs1535532 | 0.002 | 0.954 |  | 0.009 | 0.301 |  | -0.006 | 0.414 |  | 1.02 (0.98, 1.07) | 0.278 |
| rs1902272 | 0.038 | 0.435 |  | 0.001 | 0.946 |  | -0.003 | 0.668 |  | 1.01 (0.96, 1.07) | 0.599 |
| rs277143 | -0.01 | 0.802 |  | 0.001 | 0.875 |  | -0.005 | 0.455 |  | 1.03 (0.98, 1.07) | 0.237 |
| rs1671966 | 0.014 | 0.75 |  | 0.005 | 0.553 |  | -0.006 | 0.43 |  | 1.02 (0.98, 1.07) | 0.369 |
| rs1327981 | -0.037 | 0.373 |  | 0 | 0.994 |  | -0.004 | 0.533 |  | 1.01 (0.97, 1.06) | 0.513 |
| rs1327980 | -0.038 | 0.355 |  | 0 | 0.965 |  | -0.005 | 0.433 |  | 1.02 (0.98, 1.06) | 0.391 |
| rs9603395 | 0.01 | 0.819 |  | 0.006 | 0.56 |  | -0.007 | 0.311 |  | 1.01 (0.97, 1.06) | 0.554 |
| rs2025908 | -0.012 | 0.796 |  | 0.002 | 0.817 |  | -0.01 | 0.17 |  | 1.02 (0.97, 1.07) | 0.464 |
| rs1571303 | -0.024 | 0.641 |  | 0.002 | 0.86 |  | -0.004 | 0.673 |  | 0.99 (0.94, 1.04) | 0.737 |
| rs569016 | -0.047 | 0.334 |  | -0.009 | 0.408 |  | -0.002 | 0.841 |  | 0.99 (0.94, 1.04) | 0.752 |
| rs587961 | 0.001 | 0.988 |  | -0.006 | 0.537 |  | 0.01 | 0.166 |  | 1.00 (0.96, 1.05) | 0.999 |
| rs2137320 | 0 | 0.995 |  | -0.014 | 0.124 |  | 0.008 | 0.213 |  | 1.01 (0.97, 1.05) | 0.684 |
| rs4980379 | 0.005 | 0.909 |  | -0.015 | 0.11 |  | 0.011 | 0.137 |  | 0.99 (0.95, 1.04) | 0.674 |
| rs661348 | -0.023 | 0.605 |  | -0.017 | 0.066 |  | 0.011 | 0.123 |  | 1.01 (0.97, 1.06) | 0.581 |
| PRS | -0.002 | 0.731 |  | 0 | 0.882 |  | 0 | 0.771 |  | 1.00 (1.00, 1.01) | 0.326 |

Age, sex, PCA10, UK Biobank assessment centre and genotyping batch were included as covariates in the regression analyses.

SNP, single nucleotide polymorphisms; PRS, polygenic risk score. PA, primary aldosteronism. LDL-C, low density lipoprotein cholesterol.

**Additional Table 4. Description of GWAS consortiums used for each phenotype**

| Contribution | Trait | Author (year) | Sample size | | Number of SNPs | Population | PMID |
| --- | --- | --- | --- | --- | --- | --- | --- |
|  |  |  | Cases | Controls |  |  |  |
| Exposure | Primary aldosteronism | Edith Le Floch (2022) | 562 | 950 | 1,048,575 | European | 36057693 |
| Outcome | Peripheral angiopathy in diseases classified elsewhere | Jian Yang (2021) | 227 | 456,121 | 10,755,904 | European | 34737426 |
| Outcome | Gangrene | Jian Yang (2021) | 116 | 456,232 | 10,755,904 | European | 34737426 |

**Additional table 5. Effect of each SNP on LEAD events**

| **SNP** | **LEAD** | | |  | **Gangrene** | | |
| --- | --- | --- | --- | --- | --- | --- | --- |
|  | **BETA** | **SE** | **P value** |  | **BETA** | **SE** | **P value** |
| rs1200470 | 0.226 | 0.195 | 0.248 |  | 0.589 | 0.274 | 0.031 |
| rs12080303 | -0.051 | 0.138 | 0.712 |  | -0.08 | 0.193 | 0.677 |
| rs1327980 | 0.451 | 0.182 | 0.013 |  | 0.608 | 0.254 | 0.017 |
| rs1327981 | 0.446 | 0.186 | 0.016 |  | 0.624 | 0.26 | 0.017 |
| rs1535532 | 0.158 | 0.192 | 0.41 |  | 0.615 | 0.268 | 0.022 |
| rs1571303 | 0.442 | 0.194 | 0.023 |  | 0.509 | 0.271 | 0.06 |
| rs1671966 | 0.392 | 0.16 | 0.014 |  | 0.607 | 0.224 | 0.007 |
| rs1869799 | 0.128 | 0.206 | 0.534 |  | 0.832 | 0.289 | 0.004 |
| rs1902272 | 0.179 | 0.23 | 0.438 |  | 0.539 | 0.321 | 0.093 |
| rs2025908 | 0.438 | 0.178 | 0.014 |  | 0.579 | 0.249 | 0.02 |
| rs2137320 |  |  |  |  | -0.205 | 0.327 | 0.530 |
| rs277129 | 0.175 | 0.19 | 0.356 |  | 0.905 | 0.267 | 0.001 |
| rs277143 | 0.214 | 0.193 | 0.267 |  | 0.608 | 0.270 | 0.024 |
| rs284277 | -0.161 | 0.205 | 0.433 |  | -0.094 | 0.287 | 0.743 |
| rs4980379 |  |  |  |  | 0.099 | 0.293 | 0.736 |
| rs563854 | 0.214 | 0.197 | 0.279 |  | 0.637 | 0.277 | 0.021 |
| rs569016 |  |  |  |  | 0.490 | 0.192 | 0.011 |
| rs587961 |  |  |  |  | 0.589 | 0.331 | 0.075 |
| rs661348 |  |  |  |  | 0.415 | 0.344 | 0.227 |
| rs6679531 | -0.05 | 0.132 | 0.706 |  |  |  |  |
| rs880315 | -0.06 | 0.217 | 0.784 |  | 0.142 | 0.304 | 0.64 |
| rs9603395 | 0.486 | 0.177 | 0.006 |  | 0.403 | 0.246 | 0.101 |

LEAD: lower extremity arterial disease; SE, standard error; SNP, single nucleotide polymorphism

**Additional table 6. Tests of pleiotropy and heterogeneity for Mendelian randomization between primary aldosteronism and LEAD events.**

| Outcome | Pleiotropy | |  | Heterogeneity | | |
| --- | --- | --- | --- | --- | --- | --- |
|  | Egger intercept P valure | Global test P value |  | Method | Q | P value |
| LEAD (remove rs569016) | 0.194 | 0.144 |  | MR Egger | 20.223 | 0.164 |
|  |  |  |  | Inverse variance weighted | 22.722 | 0.121 |
| Gangrene (remove rs6679531) | 0.602 | 0.239 |  | MR Egger | 25.352 | 0.149 |
|  |  |  |  | Inverse variance weighted | 25.727 | 0.175 |

Q statistics refer to Cochran’s Q for IVW and Rücker’s Q for MR Egger, a test of heterogeneity or dispersion in the SNP effects.

LEAD: lower extremity arterial disease; IVW, inverse variance weighted.


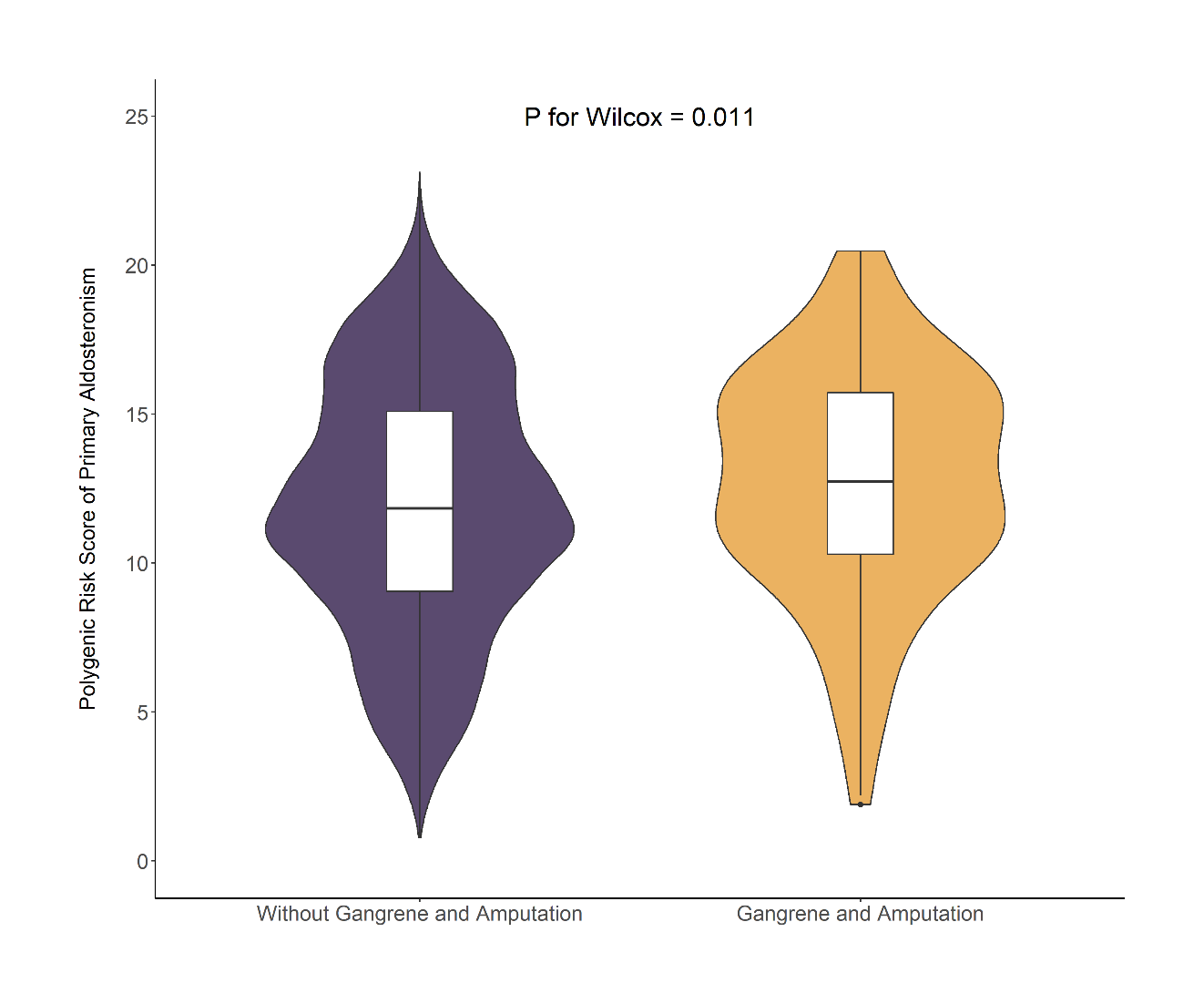


**Additional Figure 1. The PA PRS between diabetes patients with gangrene or amputation and patients without gangrene or amputation.**

PA: primary aldosteronism. PRS: polygenic risk score


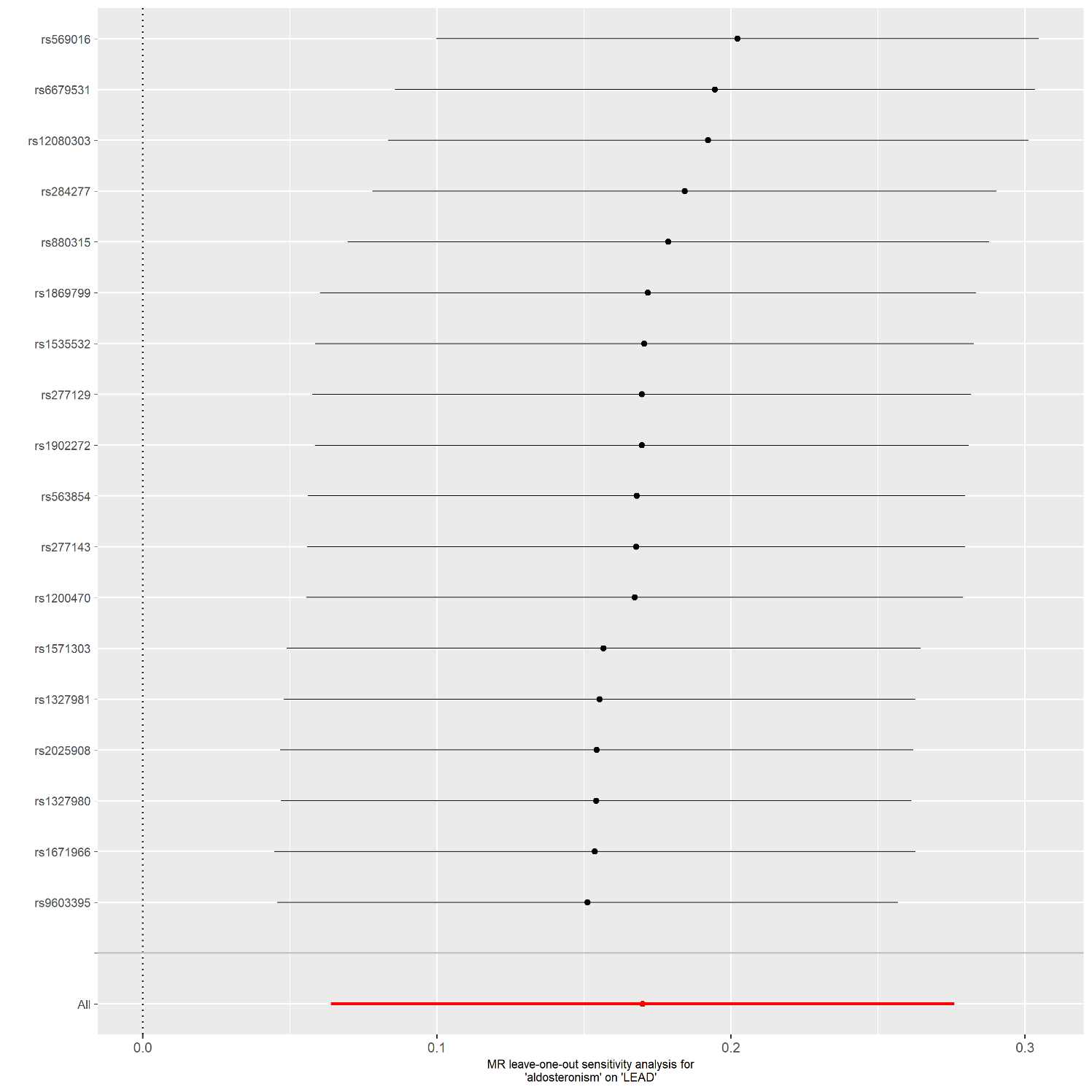


**Additional Figure 2. Leave-one-out sensitivity analysis for mendelian randomization between primary aldosteronism and lower extremity arterial disease.**


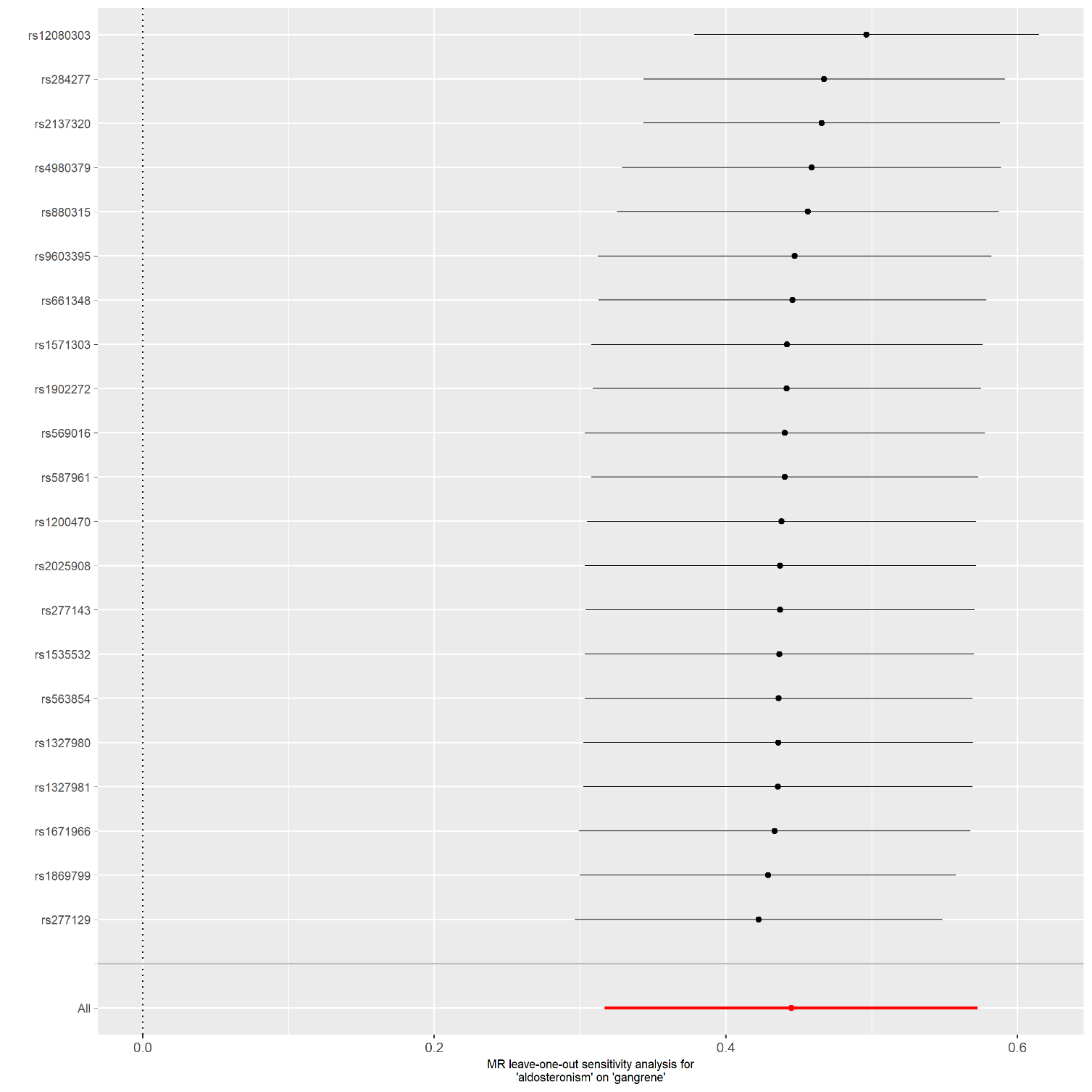


**Additional Figure 3. Leave-one-out sensitivity analysis for mendelian randomization between primary aldosteronism and gangrene.**


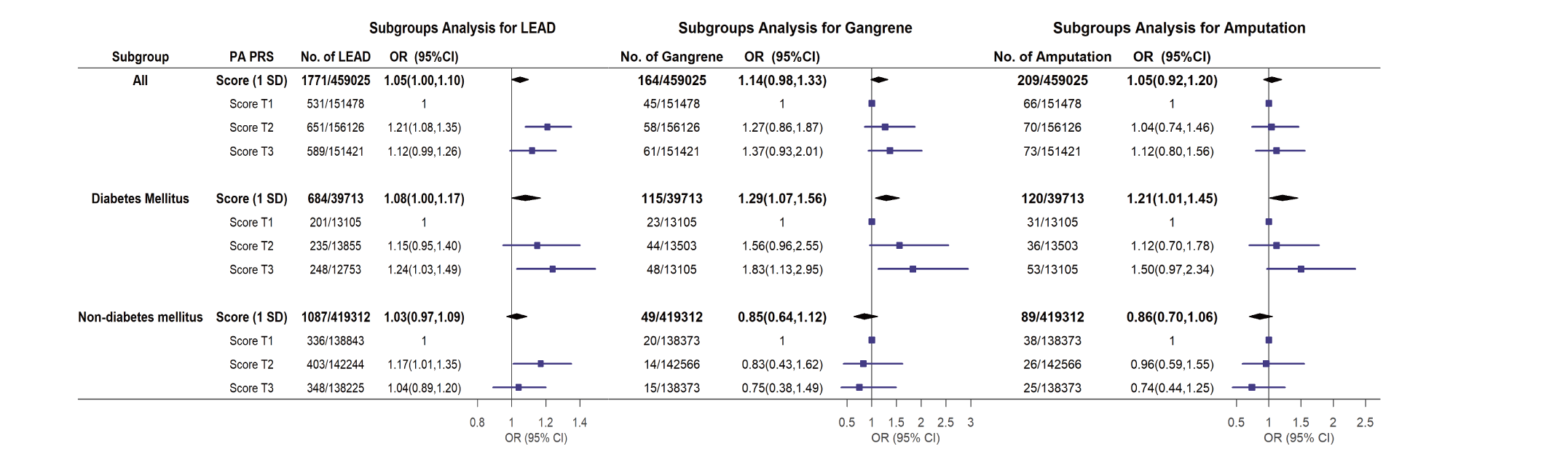


**Additional Figure 4. The regression analysis compared the association between a PA PRS based on 22 SNPs and LEAD, and LEAD-related outcomes to the association between a PA PRS that excluded 7 specific SNPs (rs284277, rs880315, rs9603395, rs587961, rs2137320, rs4980379, rs661348) and LEAD and its related outcomes.**

PA: primary aldosteronism. PRS: polygenic risk score; LEAD: lower extremity arterial diseas
